# Post-Hatch Starvation in Black Soldier Fly Larvae: Implications for Growth, Nutritional Quality, and Multi-Omics Profiles Across Rearing Substrates

**DOI:** 10.3390/microorganisms14102142

**Published:** 2026-09-23

**Authors:** Jianlai Guo, Minghui Jiao, Yuting Li, Huibin Shi, Jun Li, Xiangbo Ji, Weixian Zhang, Kai Quan, Yibao Jiang, Kun Liu

**Affiliations:** 1 Henan Key Laboratory of Unconventional Feed Resources Innovative Utilization, College of Animal Science and Technology, Henan University of Animal Husbandry and Economy, Zhengzhou 450046, China; 13837147053@163.com (J.G.); huibinshi0715@163.com (H.S.); lijun.nn@163.com (J.L.); 80104@hnuahe.edu.cn (X.J.); zhangwx126@126.com (W.Z.); quankai1115@163.com (K.Q.); 2 College of Animal Science and Technology, Henan Agricultural University, Zhengzhou 450046, China; m18239340797@163.com (M.J.); liyuting018@163.com (Y.L.)

**Keywords:** black soldier fly, starvation duration, nutritional composition, growth performance, omics technologies

## Abstract

The black soldier fly (BSF, *Hermetia illucens* L.) has gained interest for its ability to convert organic waste into high-quality protein. The eggs of BSF are typically sold in fixed quantities, which often does not match the fluctuating waste input of processing facilities; post-hatch starvation offers a practical strategy to buffer this supply-demand mismatch. However, how post-hatch starvation affects larval development and nutrient accumulation remains unclear. After being reared to approximately 5 mg, neonate larvae were subjected to starvation periods of 0, 7, 14, and 21 days in wheat bran. After starvation, larvae were reared on two substrates: kitchen waste and chicken manure, to evaluate how starvation time influences larval nutrition, growth, microbiota and metabolome. With prolonged starvation, larvae reared on kitchen waste showed increased crude protein and total amino acids, while crude fat exhibited a biphasic pattern, decreasing initially before rising again. The proportion of C12:0 fatty acid consistently increased. In contrast, larvae reared on chicken manure showed an increase in crude fat content after 21 days, alongside decreased total amino acids and increased C12:0. After 21 days of starvation, the survival rate dropped to 68%. The final fresh body weight and length of larvae in both groups increased with prolonged starvation duration. Substrate conversion efficiency peaked at 7 days of starvation across both substrates, whereas substrate reduction rates progressively declined with extended starvation, reaching the lowest values at 21 days. Microbiota analysis of the larval intestines revealed that the gut bacterial community underwent selective remodeling in response to substrate type and starvation duration. Specifically, kitchen waste, rich in fermentable carbohydrates and proteins, promoted an increased relative abundance of *Ignatzschineria* to decompose nitrogenous organic matter and supply energy. Conversely, chicken manure, which is higher in refractory organic carbon, enriched *Moheibacter* to degrade complex compounds such as chitin and cellulose. Metabolomic changes included enrichment in tryptophan, linoleic acid, and α-linolenic acid metabolism pathways. Ultimately, the studies revealed that 7 days is the optimal fasting duration to maximize bioconversion efficiency and larval production, while fasting for up to 14 days remains a safe preservation limit before survival rates significantly decline. These findings clarify how post-hatch starvation shapes BSF larval composition, growth, and molecular profiles, aiding the optimization of preservation duration and the enhancement of bioconversion efficiency and larval nutritional quality of BSF.

## 1. Introduction

The black soldier fly (BSF, *Hermetia illucens* L.) has gained increasing attention as a promising biological agent for converting organic waste into valuable biomass, addressing both the global protein feed shortage and the mounting problem of organic waste accumulation [1,2]. Black soldier fly larvae (BSFL) exhibit remarkable bioconversion efficiency: one kilogram of neonate larvae can process up to 25 tons of organic domestic waste per week, generating approximately 5 tons of larval biomass and 15 tons of organic fertilizer [3]. Nutritionally, BSFL contain 30.7–53.0% crude protein with a balanced amino acid profile and 8.1–47.4% fat, making them a viable alternative to soybean meal and fishmeal in animal diets [4,5]. Feeding trials have confirmed positive effects of BSFL inclusion in diets for poultry, swine, aquaculture species, and pets, including improved growth performance, intestinal health, and immunity [6,7,8,9].

Despite these benefits, a practical bottleneck exists in the current supply chain. BSF eggs are typically sold in fixed-quantity containers (e.g., 50 g per bottle), whereas waste processing facilities face daily fluctuations in organic waste volumes. This mismatch often leads to either a shortage of larvae for waste treatment or an excess of larvae that cannot be fully utilized, compromising operational efficiency. To reconcile this supply–demand imbalance, extending the preservation time of BSF after hatching—specifically at the larval stage—has emerged as a logical and cost-effective strategy.

Current preservation studies have largely focused on the egg stage, which is delicate and requires stringent conditions to maintain viability. Appropriate moisture content is critical, as water deficit impairs growth and immune function, although moderate drought may be tolerated [10,11]. Temperature also plays a key role. Resconi et al. [12] demonstrated that eggs of BSF can be stored at low temperatures (e.g., 10 °C) for up to about 6 days, but lower temperatures (6 °C) significantly shorten survival. These approaches demand precise environmental control and often require refrigeration equipment, which increases production costs and may adversely affect larval vigor. In contrast, preserving larvae after hatching offers a more accessible alternative: larvae can be maintained under the same ambient temperature (30 °C) used for normal rearing, without the need for cooling devices. Moreover, because larvae are already housed in the bran substrate used for hatching, no additional nutrients are required during starvation—only periodic water spraying to maintain humidity. In our preliminary trials, allowing larvae to reach a body weight of approximately 5 mg before starvation significantly enhanced their tolerance to nutrient deprivation. These advantages make post-hatch larval preservation a simple, low-cost, and easily implementable strategy for production facilities.

However, the effects of different starvation durations on the subsequent performance of BSFL—including growth, nutritional composition, gut microbiota, and metabolome—remain largely unexplored. In this study, we therefore subjected BSFL (after they had been reared to ~5 mg on wheat bran) to starvation in the bran substrate for 0, 7, 14, or 21 days, with moisture maintained by daily spraying at 30 °C and 70% relative humidity. After each starvation period, the larvae were transferred to two common rearing substrates—kitchen waste and chicken manure—to evaluate their recovery and performance. Our objectives were to (i) determine the maximum starvation duration that does not compromise survival and nutritional quality, (ii) compare substrate-specific responses, and (iii) provide a science-based recommendation for practical larval preservation in BSF production systems, thereby optimizing the use of fixed-quantity egg batches and enhancing overall bioconversion efficiency.

## 2. Materials and Methods

### 2.1. Experimental Design

The experimental workflow consisted of three consecutive phases: pre-rearing, starvation preservation, and post-preservation rearing. Pre-rearing phase: The kitchen waste, chicken manure, and BSF eggs used in this experiment were all obtained from the same batch of collection at the experimental station of Henan Agricultural University, located at 34°79′ N, 113°82′ E (Zhengzhou, Henan, China). Kitchen waste was composed mainly of plant-derived residues (rice, noodles, vegetables, and fruit peels), while animal-derived residues (meat, bones, and eggs) accounted for a small and variable proportion. Chicken manure contained feed residues, feathers, and excreta. The substrates (kitchen waste and chicken manure) were stored frozen until use. The BSF eggs were incubated directly in a controlled climate chamber(Shanghai Yiheng Scientific Instruments Co., Ltd., Shanghai, China) (30 °C, 70% humidity). After hatching, the neonates were reared on wheat bran (mixed with water at a 1:2 ratio, *w*/*v*) until they reached an average body weight of approximately 5 mg. Starvation preservation phase: Once the larvae reached approximately 5 mg, they were subjected to starvation in the original wheat bran used for hatching, with no supplemental feed added. The starvation conditions were as follows: temperature 30 ± 0.5 °C, relative humidity 70 ± 5%, and bran moisture maintained at 65–70% (*w*/*w*) by spraying sterile distilled water daily or as needed. The larvae were kept in ventilated plastic containers under the same climate-chamber conditions as the pre-rearing phase. Based on the starvation duration, the larvae were divided into four groups: 0, 7, 14, and 21 days. Notably, the 0-day group experienced zero starvation stress and was immediately transferred to the rearing substrates; therefore, this group served as the control group for this study. To monitor survival during starvation, an additional cohort of 150 larvae was taken from the 0-day group and maintained under the same starvation conditions in 100 g of wheat bran (five replicates per treatment). Viable larvae were counted at 7, 14, and 21 days, and the survival percentages at each time point were calculated accordingly. Post-preservation rearing phase: After each starvation period, the larvae from each group were immediately transferred to two common rearing substrates: kitchen waste and chicken manure. This generated eight experimental groups in total: K0, K7, K14, K21 (kitchen waste) and M0, M7, M14, M21 (chicken manure). Each treatment comprised 5 replicates, with each replicate containing 1500 larvae per 1 kg of substrate. To prepare the required larval quantity per replicate, the larval density per gram was determined, and the corresponding mass for 1500 larvae was then calculated. At the end of the rearing phase, the contents of each replicate were transferred to a stainless-steel sieve with a 2.0 mm mesh. The sieve was gently shaken for approximately 2 min. Larvae retained on the sieve were collected with clean forceps, while frass particles passing through the sieve were collected from the tray beneath. Larvae adhering to the container walls, lid, or sieve were manually removed. The separated larvae and frass were then weighed separately. Larvae from each replicate were then rinsed under running water, immersed in 75% ethanol for surface sterilization, rinsed with sterile water, and surface-dried with sterile filter paper. A portion of the BSFL intestines was dissected and stored at −80 °C for 16S rRNA sequencing and metabolomic profiling, while the remaining larval biomass was kept at −20 °C for subsequent nutritional analyses.

### 2.2. Nutritional Components of Larvae

The conventional nutritional composition of the larvae was analyzed following the procedure described by Liu et al. [13]. Crude protein content was determined using the Kjeldahl nitrogen method during the digestion process; crude fat was measured by Soxhlet extraction with petroleum ether as the solvent; moisture and dry matter contents were calculated based on weight loss after oven-drying at 105 °C for 4 h; ash content was determined by combustion in a muffle furnace (Shanghai Yiheng Scientific Instruments Co., Ltd., Shanghai, China) at 550 °C until constant weight was achieved.

Amino acid analysis was performed following the methodology described by Zulkifli et al. [14]. Samples were first subjected to acid hydrolysis using hydrochloric acid. A portion of the resulting hydrolysate was then derivatized with AccQ-Fluor reagent, using a standard amino acid as an internal standard. The amino acid derivatives were analyzed using HPLC (Alliance e2695, Waters Corporation, Milford, MA, USA) with a fluorescence detector (2475, Waters Corporation, Milford, MA, USA) and separated on a Waters AccQ-Tag Ultra C18 column (3.9 mm × 150 mm, 3.5 µm). Quantification of individual amino acids was conducted via external standard calibration with α-aminobutyric acid (AABA) to correct for potential variability in derivatization efficiency between samples.

Fatty acid composition was determined according to the method established by Zulkifli et al. [14]. Briefly, lipids were first saponified by refluxing with 1 M potassium hydroxide in 95% ethanol, followed by extraction using a hexane-diethyl ether mixed solvent. The organic phase was then centrifuged at 940× *g* for 10 min and subsequently washed with distilled water. Free fatty acids were recovered by rotary evaporation under reduced pressure. For gas chromatographic analysis, fatty acid methyl esters (FAMEs) were prepared by transesterification: the extracted oil was dissolved in a mixture of toluene and 1% sulfuric acid in methanol and allowed to react overnight. The reaction mixture was then neutralized with a 5% aqueous sodium chloride solution, and the resulting FAMEs were washed with a 2% potassium carbonate solution and dried by passing through a short column of anhydrous sodium sulfate. Finally, the purified FAMEs were diluted in n-hexane and analyzed by gas chromatography using an Agilent HP-INNOWAX capillary column (30 m × 0.25 mm, 0.25 µm, Agilent Technologies, Santa Clara, CA, USA).

### 2.3. Growth Performance of Larvae

During the rearing period, larval body weight was monitored following the sampling protocol described by Deruytter et al. [15]. Briefly, subsamples were collected from multiple locations within each replicate, pooled to obtain at least 100 larvae, rinsed with tap water, surface-dried with paper towels, and weighed collectively to calculate the average wet weight per larva. Additionally, 50 larvae were randomly sampled from each group for average body length measurement using a Vernier caliper (Wuxi Sida Measuring Tools Co., Ltd., Wuxi, China), as described by Zulkifli et al. [14]. At the conclusion of the experiment, samples of larval frass, substrate, and larvae were collected and oven-dried at 105 °C until reaching a constant weight, in order to calculate moisture content for each sample. Based on these measurements, the following parameters were calculated according to predefined formulas:

(1) $$\mathrm{SR}\;(\%)\;=\;\frac{\mathrm{SL}}{\mathrm{IL}}\;\times \;100$$

(2) $$\mathrm{LP}\;(\%)\;=\;\frac{{\mathrm{LW}}_{w}}{\;{\mathrm{SW}}_{w}\;}\;\times \;100$$

(3) $${\mathrm{SR}}_{w}\;\left(\%\right)\;=\;\frac{\left(\mathrm{SW}-\mathrm{FW}\right)}{\mathrm{SW}}\;\times \;100$$

(4) $${\mathrm{SC}}_{w}\;(\%)\;=\;\frac{\mathrm{BW}}{\left(\mathrm{SW}-\mathrm{FW}\right)}\;\times \;100$$

(5) $${\mathrm{SR}}_{D}\;\left(\%\right)\;=\;\frac{\left(\mathrm{SDM}-\mathrm{FDM}\right)}{\mathrm{SDM}}\;\times \;100$$

(6) $${\mathrm{SC}}_{D}\;(\%)\;=\;\frac{\mathrm{BDM}}{\left(\mathrm{SDM}-\mathrm{FDM}\right)}\;\times \;100$$

where SR: survival ratio; SL: number of surviving larvae; IL: initial larvae number; LP: larval production rate; LW_W_: wet weight of larvae; SW_W_: wet weight of substrate; SR_W_: substrate fresh weight reduction; SW: initial substrate fresh weight; FW: frass fresh weight; SC_W_: substrate fresh weight conversion; BW: insect body fresh weight; SR_D_: substrate dry matter reduction; SDM: initial substrate dry matter; FDM: frass dry matter; SC_D_: substrate dry matter conversion; BDM: insect body dry matter.

### 2.4. Microbiota Analysis

DNA samples were extracted using the E.Z.N.A.^®^ gut DNA Kit (Omega Bio-tek, Norcross, GA, USA), with concentration and purity determined using a NanoDrop 2000 UV-vis spectrophotometer (Thermo Scientific, Wilmington, DE, USA). The V3-V4 region of bacterial 16S rRNA was amplified with primers 338F (5′-ACTCCTACGGGAGGCAGCAG-3′) and 806R (5′-GGACTACHVGGGTWTCTAAT-3′). PCR was carried out under the following conditions: 95 °C for 3 min; 27 cycles of 95 °C for 30 s, 55 °C for 30 s, and 72 °C for 45 s; followed by a final extension at 72 °C for 10 min on an ABI GeneAmp^®^ 9700 PCR thermocycler (Applied Biosystems, Foster City, CA, USA). The products were separated and purified using the AxyPrep DNA Gel Extraction Kit (Axygen Biosciences, Union City, CA, USA). Purified amplicons were subjected to paired-end sequencing on the Illumina MiSeq PE300 platforms at Majorbio Bio-Pharm Technology Co., Ltd. The raw 16S rRNA gene sequencing reads were demultiplexed, quality-filtered using fastp version 0.20.0 and merged using FLASH version 1.2.7 with the following criteria: (i) the 300 bp reads were truncated at any site receiving an average quality score of <20 over a 50 bp sliding window, and the truncated reads shorter than 50 bp were discarded; reads containing ambiguous characters were also discarded; (ii) only overlapping sequences longer than 10 bp were assembled according to their overlapped sequence, with a maximum mismatch ratio of 0.2; reads that could not be assembled were discarded [16,17]. Operational taxonomic units (OTUs) were clustered with a 97% similarity cutoff using UPARSE version 7.1, and chimeric sequences were identified and removed [18]. The taxonomy of each OTU representative sequence was assigned using the RDP Classifier version 2.2 against the SILVA 16S rRNA database version 138 with a confidence threshold of 0.7 [19,20,21].

### 2.5. Metabolomics Analysis

Samples (50 mg) were homogenized in methanol: water (4:1) with internal standard (L-2-chlorophenylalanine) using cryogenic grinding (−10 °C) and ultrasonication (5 °C). QC samples (pooled from all samples) were injected every 5–15 samples for system stability monitoring. Analysis was performed on a Thermo UHPLC-Q Exactive HF-X system at Majorbio Bio-Pharm Technology Co., Ltd. Using an ACQUITY HSS T3 column (100 mm × 2.1 mm, 1.8 µm, Waters Corporation, Milford, MA, USA) with 0.1% formic acid in water (A) and acetonitrile: isopropanol (47.5:47.5) with 0.1% formic acid (B). The raw LC-MS data were processed using Progenesis QI software (version 3.0, Waters Corporation, Milford, MA, USA). Metabolites were then identified by matching MS and MS/MS spectral information against public databases, including HMDB and METLIN, as well as a self-built library. The resulting datasets were uploaded to the Majorbio Cloud Platform for further preprocessing. Subsequently, Principal Component Analysis (PCA) and Orthogonal Partial Least Squares Discriminant Analysis (OPLS-DA) were performed using the “ropls” package (v1.6.2) in R. The stability of the OPLS-DA model was validated using seven-fold cross-validation. Significantly different metabolites were identified based on a variable importance in projection VIP > 1.0 from the OPLS-DA model and a *p* < 0.05 from a Student’s *t*-test. Metabolite pathways were annotated using the KEGG database. Finally, pathway enrichment analysis was performed using Fisher’s exact test with the “scipy.stats” package in Python (version 1.7.3, NumFOCUS, Austin, TX, USA) to identify significantly enriched biological pathways.

### 2.6. Statistical Analysis

All data were organized and managed using Microsoft Excel. Statistical analysis was conducted with IBM SPSS Statistics (version 26.0). Prior to one-way analysis of variance (ANOVA), normality of residuals was assessed using the Shapiro–Wilk test, and homogeneity of variances was evaluated using Levene’s test. When these assumptions were met, one-way ANOVA was performed, followed by Duncan’s multiple range test for post hoc comparisons. The statistical unit was the replicate (bucket containing the larvae), with *n* = 5 replicates per treatment group for nutritional, growth, and production parameters. For microbiome and metabolomics analyses, *n* = 5 replicates per treatment were also used, with the exception that some samples were pooled for QC purposes as described in the respective sections. In the tables, results are presented as treatment means alongside the pooled standard error of the mean (SEM) derived from the ANOVA model. In the text and figures, data may be represented as mean ± standard deviation (SD) where appropriate. Significance levels were defined as follows: * *p* < 0.05 was considered statistically significant, ** *p* < 0.01 as highly significant, and *** *p* < 0.001 as extremely significant. All figures were generated using GraphPad Prism (version 10).

## 3. Results

### 3.1. Impact of Starvation on Nutritional Composition of BSFL

The proximate composition of the two substrates is summarized in Table 1. Specifically, the kitchen waste contained 77.71% moisture, 8.15% ash, 34.62% crude protein, and 13.70% crude lipid on a dry matter basis. In comparison, the chicken manure exhibited a similar moisture content (76.05%) and comparable crude protein level (31.05%), but had a significantly higher ash content (36.79%) and a markedly lower crude lipid content (7.45%) (*p* < 0.05). Across both kitchen waste and chicken manure groups, ash and fat contents, as shown in Table 2 and Table 3, exhibited similar trends: ash content initially increased and then decreased, peaking at day 14, whereas fat content showed an opposite pattern, decreasing first and then recovering by day 21. In contrast, moisture and protein contents showed substrate-specific responses. In the kitchen waste group, crude protein progressively increased with prolonged starvation, reaching the highest level at day 21 (46.60%), while moisture was higher at days 7 and 21. In the chicken manure group, no significant changes were observed in either moisture or crude protein across starvation durations.

The amino acid profiles of the raw substrates are given in Table 4, demonstrating that kitchen waste possessed significantly higher contents of total amino acids (27.25%) and essential amino acids (11.86%) compared to chicken manure (9.58% and 3.35%, respectively) (*p* < 0.05). The larval amino acid data are detailed in Table 5 and Table 6. The total (TAA), essential (EAA), and non-essential (NEAA) amino acid contents showed opposite trends between the two substrates. In kitchen waste-reared larvae, TAA, EAA, and NEAA all increased with starvation, peaking at day 21 (34.98%, 16.01%, and 18.94%, respectively), with notable rises in leucine, lysine, methionine, and valine among EAAs, and in aspartic acid and alanine among NEAAs. Conversely, in chicken manure-reared larvae, all three categories declined progressively, reaching their lowest values at day 21, with marked reductions in phenylalanine, threonine, arginine, glycine, and glutamic acid. These opposite trends were consistent with the changes observed for crude protein.

The fatty acid composition of the raw substrates is compiled in Table 7. Notably, kitchen waste was richer in monounsaturated fatty acids (MUFA, 33.47%), whereas chicken manure contained a significantly higher proportion of saturated fatty acids (SFA, 33.62%) (*p* < 0.05). Table 8 and Table 9 present the corresponding larval fatty acid responses. In kitchen waste-reared larvae, SFA (C12:0, C16:0) progressively increased with starvation, driven largely by a marked rise in C12:0 (lauric acid) from 18.39% to 26.10%; MUFA (C18:1n-9c) remained relatively stable, while PUFA C18:2n-6c) decreased at day 21. In chicken manure-reared larvae, SFA (C12:0, C16:0) peaked at days 7–14 and then declined; MUFA (C18:1n-9c) gradually decreased over time, whereas PUFA (C18:2n-6c) showed a late increase at day 21. Notably, C12:0 content was consistently higher in kitchen waste-reared larvae (18.39–26.10%) than in chicken manure-reared larvae (3.36–9.38%) across all time points. Yet in both substrate groups, the proportion of C12:0 consistently increased with prolonged starvation.

### 3.2. Growth and Bioconversion Performance in Response to Starvation

Figure 1 and Figure 2 illustrate the changes in body weight and length of BSFL over the rearing period. On both substrates, final body weight and length generally increased with extended starvation duration. On kitchen waste, final weight rose progressively with starvation time, with the K21 group attaining the highest value. On chicken manure, the M21 group also reached the greatest final weight, although the M7 group showed a transient growth lag relative to the non-starved control. Similarly, final body length increased with starvation on both substrates: the K7 and K21 groups performed best on kitchen waste, while the M14 and M21 groups showed the greatest elongation on chicken manure. Overall, despite minor substrate-specific fluctuations, both weight and length tended to increase with prolonged starvation, suggesting a compensatory growth response upon refeeding.

Table 10 and Table 11 summarize the biomass production parameters for kitchen waste and chicken manure groups, respectively. Survival rate (SR) remained above 95% after 7 and 14 days of starvation but dropped sharply to 67.60% at 21 days. Larval production (LP) was highest at day 7 in both substrates and declined significantly from day 14 onward, mirroring the mortality trend. Substrate reduction rates, on both fresh weight (SR_W_) and dry matter (SR_D_) bases, progressively decreased with prolonged starvation across both substrates. In contrast, substrate conversion efficiencies (SC_W_ and SC_D_) showed different patterns: SC_W_ was highest at day 7 in the kitchen waste group but peaked at day 21 in the chicken manure group. SC_D_ peaked at day 7 in both groups and remained relatively high at day 21, particularly in the chicken manure group. These results indicate that starvation up to 14 days preserves conversion performance, while 21-day starvation compromises overall output.

### 3.3. Shifts in Gut Microbiota Under Starvation

As shown in Figure 3, starvation stress significantly altered the gut microbial community of kitchen waste-reared larvae. Beta-diversity analysis revealed distinct clustering patterns among starvation groups (K0, K7, K14, K21) with minimal intra-group variation, except for substantial inter-group divergence in K21 (Figure 3A). Shannon diversity was higher in K0 and K14 but lower in K7 and K21 (Figure 3B). A total of 123 shared OTUs were identified, with K21 harboring the most unique OTUs (Figure 3C). At the phylum level, *Actinomycetota*, *Pseudomonadota*, *Bacteroidota*, and *Bacillota* dominated, with *Bacteroidota* and *Actinomycetota* notably enriched in K14 (Figure 3D). Genus-level composition was primarily *Enterococcus*, *Ignatzschineria*, and *Morganella*; compared to K0, *Morganella* decreased in K7, an unclassified *Dysgonomonadaceae* increased in K14, and K21 showed reduced *Enterococcus* but increased *Ignatzschineria* (Figure 3E).

Beta-diversity analysis revealed distinct clustering with minimal intra-group variation except for M7, which showed substantial inter-group heterogeneity (Figure 4A). Shannon diversity was higher in M0 but lower in M7 and M21 (Figure 4B). A large number of shared OTUs were found, with M21 harboring the most unique OTUs (Figure 4C). At the phylum level, *Proteobacteria*, *Firmicutes*, and *Bacteroidetes* dominated, with compositional shifts across time points (Figure 4D). At the genus level, in M21, *Moheibacter* increased significantly while *Dysgonomonas* and *Enterococcus* declined markedly, indicating a starvation-driven shift in microbial dominance (Figure 4E).

### 3.4. Metabolic Alterations Induced by Starvation

Principal component analysis (PCA) revealed minimal intra-group variation among the four K groups, and differentially accumulated metabolites were classified into 20 KEGG pathways (Figure 5A). Venn diagram visualization showed 6714 common metabolites, with the K21 group having the highest number of unique metabolites (55 compounds) (Figure 5B). KEGG topological analysis indicated that linoleic acid, α-linolenic acid, and tryptophan metabolism were the most significantly altered pathways; tryptophan metabolism was notably affected in K7 and K21, while α-linolenic acid metabolism was perturbed in K14 and K21 (Figure 5C,E,G). VIP analysis for K7 vs. K0 revealed upregulation of quinoline-4,8-diol, retinal, tryptamine, 4-hydroxybenzaldehyde, and L-formylkynurenine, and downregulation of furfural and 11-HPODE (Figure 5D). VIP analysis for K14 vs. K0 showed upregulation of γ-linolenic acid, UDP-N-acetylglucosamine, and retinal, and downregulation of 9-oxo-nonanoic acid, 11-HPODE, 13(S)-hydroperoxylinolenic acid, jasmonic acid, and methyl jasmonate (Figure 5F). VIP analysis for K21 vs. K0 demonstrated upregulation of tryptamine, anthranilic acid, citrulline, and argininosuccinic acid, and downregulation of methyl jasmonate, thymidine, thymidine 3′,5′-cyclic monophosphate, and 13(S)-hydroperoxylinolenic acid (Figure 5H).

For the chicken manure groups, PCA showed clear segregation of M21 from the others, and differentially expressed metabolites were also categorized into 20 KEGG pathways (Figure 6A). A total of 6418 metabolites were common to all groups, and M21 had the highest number of unique metabolites (118 compounds) (Figure 6B). KEGG topological analysis consistently identified tryptophan metabolism as a core altered pathway across all M groups; arginine biosynthesis was significantly perturbed in M7 and M14, while M21 shifted towards linoleic acid and tyrosine metabolism (Figure 6C,E,G). VIP analysis for M7 vs. M0 showed downregulation of tryptamine, N-acetylputrescine, citrulline, and serotonin (Figure 6D). VIP analysis for M14 vs. M0 revealed upregulation of N-acetylserotonin, N-formylanthranilic acid, tryptamine, nicotinamide, serotonin, and anthranilic acid, and downregulation of L-aspartate-semialdehyde, melatonin, and L-cystathionine (Figure 6F). VIP analysis for M21 vs. M0 indicated upregulation of serotonin, tryptamine, and 8(R)-HpODE, and downregulation of 4-coumaroyl-CoA, melatonin, N-formylanthranilic acid, and anthranilic acid (Figure 6H).

## 4. Discussion

### 4.1. Nutritional Components of BSFL

The relative proportion of crude protein in BSFL reared on kitchen waste increased with prolonged starvation periods prior to refeeding. It is important to clarify that starvation itself does not generate protein; rather, this increase is driven by a compensatory biological response combined with metabolic substrate partitioning. Prolonged nutrient deprivation triggers a compensatory assimilation mechanism. When these starved larvae are subsequently reared on highly bioaccessible kitchen waste, they efficiently mobilize the abundant dietary nitrogen toward protein synthesis for rapid tissue repair. Furthermore, as lipid reserves are heavily catabolized for energy during early starvation, the relative percentage of protein mathematically rises. In contrast, larvae raised on chicken manure displayed a decrease in protein proportion. These opposite trends reflect differences in substrate nitrogen composition: kitchen waste contains higher levels of readily assimilable nitrogenous compounds, while chicken manure has a substantial proportion of non-protein nitrogen (e.g., uric acid) that is less efficiently utilized by BSFL [22]. Moreover, the protein decline in chicken manure-reared larvae suggests that prolonged starvation actively impairs the utilization of such poor-quality nitrogen sources [23]. The crude fat content in both substrates declined initially and then increased, reaching its lowest point at day 7. This pattern may result from accelerated lipid catabolism to sustain basal metabolic demands during short-term starvation, consistent with Lee et al. [24]. The subsequent increase in crude fat at 21 days may be associated with density-dependent effects: prolonged starvation elevated larval mortality, reducing population density and alleviating intraspecific competition, thereby enabling surviving individuals to access greater nutritional resources and enhance lipid accumulation. In black soldier fly, Opare et al. [25] demonstrated that low larval density resulted in larger prepupal mass, higher survival, greater fat content, and shorter development time, while Dzepe et al. [26] reported that individual larval weight, body size, and survival rate were negatively correlated with rearing density. In summary, starvation duration differentially modulates protein and lipid accumulation in BSFL depending on the rearing substrate, with density-mediated competition playing a key role in lipid restoration at later time points.

Regarding specific amino acids, in BSFL reared on kitchen waste, isoleucine, leucine, lysine, and valine gradually increased with extended starvation; in contrast, phenylalanine, threonine, and arginine declined in chicken manure-reared larvae. The divergent trends between substrates may be explained by the same factors discussed above for crude protein: kitchen waste provides more readily assimilable nitrogenous substrates that support amino acid anabolism upon refeeding, whereas the non-protein nitrogen in chicken manure limits the availability of key amino acid precursors [22]. The enrichment of lysine, leucine, isoleucine, and valine in kitchen waste-reared larvae is noteworthy, as these branched-chain and limiting amino acids are known to promote growth, protein synthesis, and energy supply in insects [27,28]. Conversely, the decline of phenylalanine, threonine, and arginine in chicken manure-reared larvae may compromise cuticle sclerotization, intestinal integrity, and nitrogen balance [23,29,30], potentially contributing to the poorer performance observed on this substrate. In summary, starvation duration alters specific amino acid profiles in a substrate-dependent manner, with kitchen waste supporting progressive enrichment of growth-related amino acids, while chicken manure results in depletion of key amino acids essential for protein synthesis and development.

The consistent enrichment of C12:0 (lauric acid) with prolonged starvation across both substrates is noteworthy. Liu et al. [31] reported that C12:0 in BSFL is primarily synthesized from carbohydrates and transformed from long-chain fatty acids. Given that lauric acid possesses recognized antiviral and antibacterial properties [32], this elevation may reflect an adaptive strategy to enhance immune defense during nutrient deprivation. Notably, the substantially higher C12:0 content in kitchen waste-reared larvae (18.39–26.10%) compared to manure-reared larvae (3.36–9.38%) suggests that BSFL fed on lipid-rich substrates exhibit greater capacity for lauric acid accumulation, a trait with potential implications for producing high-value larval biomass. In the kitchen waste group, the proportions of C16:0 (palmitic acid) and C18:2n-6c (linoleic acid) decreased progressively with starvation time, which may be attributable to the increased relative proportion of C12:0. Palmitic acid serves as an energy reserve and membrane structural component [33], while linoleic acid is an essential polyunsaturated fatty acid required for cellular function and growth [34]. In the chicken manure group, C16:0 showed an initial increase followed by a decrease, whereas C18:2n-6c showed the opposite pattern. These contrasting dynamics suggest that BSFL adjust their fatty acid utilization strategies according to substrate type and starvation stress. In summary, prolonged starvation consistently enriches C12:0 in BSFL regardless of substrate, while the responses of C16:0 and C18:2n-6c are substrate-dependent, reflecting distinct metabolic adjustments in energy allocation and immune preparedness.

### 4.2. Growth Performance and Bioconversion of BSFL

Across both rearing substrates, larval weight and length showed significant increases with extended starvation time. This growth pattern may be attributed to a compensatory effect of BSFL following starvation stress, where, upon resuming feeding, larvae exhibit a heightened tendency toward lipid accumulation to mitigate potential future nutritional challenges, consequently driving increases in both body weight and length [35,36]. Furthermore, after 21 days of starvation, the substantial decline in SR reduced intraspecific competition, allowing remaining individuals access to more abundant resources, which further contributed to significant increases in larval size [37].

In this study, the survival rate remained high (95.73%) after 14 days of starvation but declined sharply to 67.60% at 21 days. In comparison, starvation led to significant survival reductions in *Trogoderma granarium* after 8 days [38] and in *Hyphantria cunea* after only 3 days [39], suggesting that BSFL possess relatively strong starvation tolerance. We attribute this improved survival to two key measures: allowing larvae to reach ~5 mg before starvation, and maintaining substrate moisture through daily watering, both of which may have enhanced larval resistance to nutrient deprivation.

Larval production (LP) peaked on day 7 (K7 and M7) and then declined with prolonged starvation. However, substrate dry matter conversion (SC_D_) in both groups remained higher at 7 and 21 days compared to the 0-day control. The higher conversion efficiency at day 7 may result from moderate starvation stress stimulating feeding activity and digestive efficiency upon refeeding [40]. At 21 days, although the substantial reduction in survival rate lowered population density and reduced intraspecific competition—thereby maintaining conversion efficiency to some extent—the insufficient number of viable individuals ultimately led to reduced overall larval production. Correspondingly, substrate reduction (both SR_W_ and SR_D_) declined with prolonged starvation across both substrates, as decreased larval survival reduced overall substrate consumption. In summary, starvation up to 14 days preserves larval survival and conversion efficiency, with day 7 representing the optimal balance between survival and productivity; 21-day starvation, despite density-mediated compensatory effects on conversion, compromises overall output due to elevated mortality.

### 4.3. Microbiota of BSFL

Starvation stress exerts a significant selective pressure on the gut microbiota, promoting the dominance of starvation-tolerant taxa while suppressing those unable to withstand nutrient deprivation [41,42]. This microbial restructuring may be further influenced by cross-feeding interactions under limited resource conditions [43]. Microbiota analysis revealed a substantial increase in unique OTUs in the K21 and M21 groups, with the latter exhibiting the highest OTU richness among all treatments. The elevated diversity and unique OTU enrichment at 21 days suggest that extended starvation may reshape the gut ecosystem by promoting specialized taxa better adapted to nutrient-limited conditions [44]. At the phylum level, the dominant taxa across both substrates were *Actinomycetota*, *Pseudomonadota*, *Bacteroidota*, and *Bacillota*. Similar phylum-level dominance has also been reported by Li et al. [45]. The relative abundances of *Bacteroidota* and *Actinomycetota* increased with prolonged starvation, consistent with Yang et al. [42], who reported that starvation for 24 and 48 h significantly altered phylum-level composition in BSFL, with increased proportions of *Actinomycetota* and *Bacteroidota* under starvation. In summary, starvation stress drives a substrate-independent shift towards increased abundance of specific phyla (*Bacteroidota* and *Actinomycetota*), while the enrichment of unique OTUs at 21 days indicates a taxonomically more specialized but potentially less stable gut community.

At the genus level, significant microbial shifts were observed in response to preservation duration. In kitchen waste-reared BSFL, *Morganella* decreased at K7, *Dysgonomonadaceae* increased at K14, and at K21, *Enterococcus* decreased while *Ignatzschineria* increased. Li et al. [46] reported that increasing the household food waste proportion in BSFL substrates enriched cellulose-degrading genera such as *Dysgonomonas* and Actinomyces. This aligns with our observation of increased *Dysgonomonadaceae* in the K14 group. However, unlike their finding of Klebsiella enrichment associated with nitrogen recovery, our K21 group showed a marked increase in *Ignatzschineria*, suggesting that prolonged starvation imposes a distinct selective pressure on the gut microbiota compared to substrate mixing alone. Members of *Dysgonomonadaceae* can ferment short-chain fatty acids to provide additional energy for larvae, enhancing hunger resistance [47], while *Ignatzschineria* is capable of decomposing nitrogenous organic matter in decaying environments [48]. These traits may collectively support host survival under prolonged nutrient deprivation by supplying nitrogen and energy. In chicken manure-reared BSFL, at the genus level, the significant enrichment of *Moheibacter* in the M21 group reveals the reconfiguration of microbial communities under starvation conditions. *Moheibacter*, belonging to the *Flavobacteriaceae* family, has previously been reported to have the ability to degrade complex organic carbon sources such as chitin and cellulose derivatives. The increase in its abundance may reflect a shift in microbial communities towards groups that are better at utilizing refractory substrates in nutrient-limited environments [49]. Furthermore, the dramatic shifts observed in the gut microbiota of kitchen waste-reared larvae suggest that kitchen waste is richer in overall nutritional composition and possesses significantly higher bioavailability and bioaccessibility than chicken manure [22]. When starved larvae are reintroduced to this nutrient-dense substrate, the highly accessible fermentable carbohydrates and crude proteins rapidly drive a dynamic microbial succession, supporting the marked enrichment of taxa like *Ignatzschineria* [47]. In contrast, chicken manure contains more ash and non-protein nitrogen, meaning its nutrients are far less bioaccessible. This poorer nutritional profile and lower bioavailability favor the proliferation of distinct taxa, such as *Moheibacter*, which are specialized in degrading refractory organic carbon to survive under nutrient-limited conditions [49].

### 4.4. Metabolomics of BSFL

Metabolomic analysis revealed that tryptophan metabolism was consistently affected across all starvation durations and both substrates, with a temporal shift from amino acid-related pathways (arginine biosynthesis) at early time points to lipid-related pathways (linoleic acid metabolism) at day 21. Tryptophan metabolism is known to participate in stress responses and physiological homeostasis [50,51], suggesting its activation as a general adaptive mechanism to starvation. The late shift towards lipid metabolism implies that when amino acid resources become limited after prolonged starvation, larvae increasingly rely on lipid catabolism for energy supply. These substrate- and time-dependent metabolic patterns indicate that BSFL adjust their metabolic priorities according to nutritional context: early starvation primarily affects amino acid metabolism, while prolonged starvation shifts towards lipid metabolism.

In chicken manure-reared larvae, metabolic profiles also varied with preservation duration. Compared with M0, the M7 group showed alterations mainly involving arginine biosynthesis and tryptophan metabolism, with changes in tryptamine, serotonin, and citrulline. The perturbation of arginine biosynthesis at day 7 may reflect an early attempt to maintain nitrogen balance and urea cycle function under nutrient deprivation [23]. The M14 group exhibited increased abundance of several tryptophan-related metabolites, including tryptamine, serotonin, and anthranilic acid. The sustained enrichment of tryptophan metabolites across days 7 to 14 suggests that the kynurenine and serotonin pathways are progressively activated as starvation extends, which may serve as an adaptive mechanism to cope with prolonged nutritional stress [50,51]. At 21 days, the M21 group showed marked changes in lipid-related metabolism, with altered abundance of metabolites involved in linoleic acid metabolism, including 8(R)-HpODE. This late shift towards lipid metabolism implies that when amino acid resources become limited after prolonged starvation, larvae may increasingly rely on lipid catabolism for energy supply. These substrate- and time-dependent metabolic patterns suggest that BSFL adjust their metabolic priorities according to nutritional context: early starvation primarily affects amino acid metabolism, while prolonged starvation shifts towards lipid metabolism. Overall, the divergent metabolic profiles between the kitchen waste and chicken manure groups are fundamentally driven by the distinct nutritional compositions and bioavailabilities of the two substrates [22]. Kitchen waste provides an abundance of readily accessible amino acids and fatty acids, allowing the previously starved larvae to efficiently mobilize these resources into pathways such as linoleic acid and α-linolenic acid metabolism for rapid lipid restoration and tissue repair. Conversely, the high levels of non-protein nitrogen and refractory carbon in chicken manure create a poor nutritional environment [22]. This forces the larvae into more severe metabolic adaptations, such as the early perturbation of arginine biosynthesis to manage nitrogen balance, before eventually having to rely on alternative lipid scavenging strategies for energy [23]. Despite these substrate-driven differences in how larvae acquire and partition nutrients, the consistent activation of tryptophan metabolism across both groups underscores its universal, substrate-independent role in mediating the physiological stress response to prolonged starvation [50,51].

Crucially, the distinct metabolic alterations and ultimate nutritional profiles of the BSFL may not merely be parallel events to the microbial shifts, but may be associated with the starvation-induced restructuring of the gut microbiome. In the kitchen waste group, the enrichment of Ignatzschineria, a taxon associated with the decomposition of nitrogenous matter, could potentially participate in processing bioaccessible dietary nitrogen and may contribute to the observed accumulation of host crude protein and essential amino acids. Conversely, in the chicken manure group, the proliferation of Moheibacter may reflect a microbial adaptation associated with the breakdown of recalcitrant carbon. However, this bacterial shift may not fully compensate for the limited availability of nitrogen, may contribute to the depletion of host amino acids, and may potentially shift the host–microbiome metabolic axis toward lipid scavenging (e.g., linoleic acid metabolism). Furthermore, the consistent perturbation of tryptophan metabolism across both substrates is consistent with possible host–microbiome crosstalk. Gut bacteria could potentially participate in tryptophan catabolism, converting it into bioactive derivatives such as tryptamine (which was significantly altered in our LC-MS data), and may contribute to the regulation of host energy homeostasis and the physiological response to starvation stress. Together, these inter-omics linkages suggest a possible functional link between the microbial taxa enriched during starvation and the subsequent metabolic pathways and bioconversion efficiency of the larvae.

### 4.5. Study Limitations and Future Perspectives

This study systematically evaluated the effects of different starvation durations (0–21 days) on the subsequent growth and development, nutritional composition, gut microbiota, and metabolites during the substrate utilization process of BSFL after hatching. The experiment selected kitchen waste and chicken manure, two commonly used substrates in Chinese insect farming systems, providing a direct and reliable reference for larvae preservation under local production conditions. However, the utilization of other types of substrates remains to be further explored. In addition, it was found that during larvae preservation, feeding them approximately 5 mg and supplementing with water spray for moisture retention can significantly improve survival rates, but preservation conditions still need to be systematically optimized. The multi-omics analyses revealed dynamic changes in gut microbial communities and metabolic pathways in response to starvation, offering valuable clues for future mechanistic studies. In particular, the functional roles of key bacterial taxa (e.g., *Dysgonomonadaceae* and *Ignatzschineria*) and characteristic metabolites (e.g., tryptophan metabolites and linoleic acid derivatives) identified in this study warrant deeper investigation to elucidate their contributions to larval performance and bioconversion efficiency. Furthermore, the microbial analysis in this study relied on 16S rRNA amplicon sequencing, which effectively captured the dominant bacterial communities driving bioconversion but did not account for eukaryotic microbes such as fungi and protozoa. Future studies utilizing shotgun metagenomics or multi-kingdom amplicon sequencing will be essential to accurately characterize the entire microbiome and fully elucidate the multi-kingdom microbial interactions under starvation stress.

## 5. Conclusions

This study systematically evaluated post-hatch starvation in BSFL to address supply-demand mismatches in waste processing facilities, providing clear answers to our initial objectives. First, the maximum starvation duration that does not compromise survival or nutritional quality is 14 days, which maintains a survival rate of >95% and preserves conversion performance; extending starvation to 21 days is unviable due to a severe reduction in survival (67.60%). Second, regarding substrate-specific responses, larvae exhibit distinctly divergent assimilation strategies upon refeeding. The high bioaccessibility of kitchen waste promotes progressive increases in crude protein and essential amino acids, alongside the enrichment of beneficial nutrient-degrading microbes (*Ignatzschineria*). In contrast, the poor bioavailability of chicken manure leads to decreased amino acid accumulation and a shift toward refractory-carbon-degrading taxa (*Moheibacter*). Finally, as a science-based recommendation for practical larval preservation: production systems can safely utilize post-hatch starvation to buffer the fluctuating waste supply against fixed-quantity egg batches. A 7-day starvation period is optimal for maximizing overall bioconversion efficiency, while 14 days serves as the strict maximum preservation limit to prevent production losses.

## Figures and Tables

**Figure 1:**
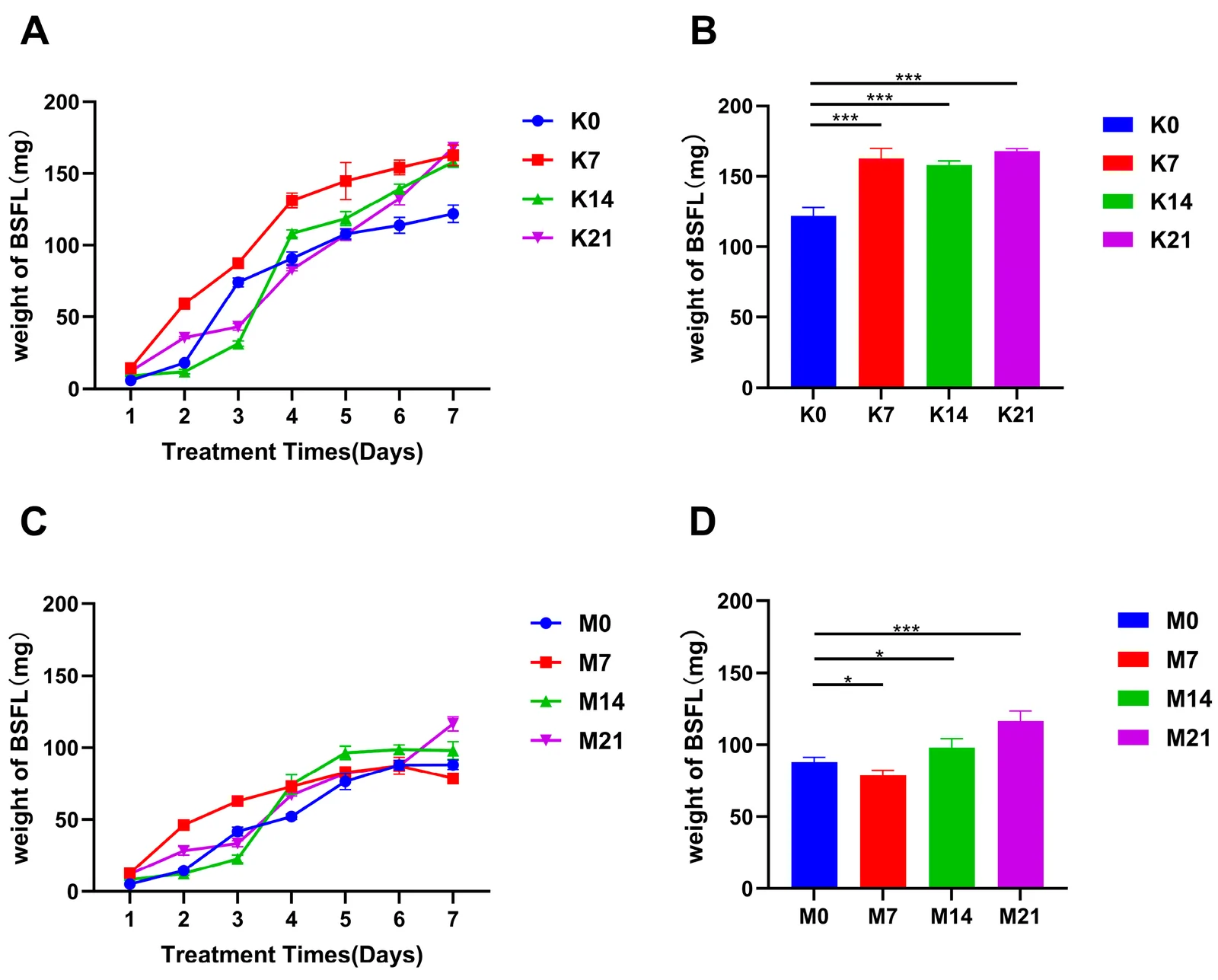
The weight of BSFL raised on kitchen waste and chicken manure after different starvation times. (**A**) Daily weight of BSFL on kitchen waste substrate. (**B**) Final day weight of BSFL on kitchen waste substrate. (**C**) Daily weight of BSFL on chicken manure substrate. (**D**) Final day weight of BSFL on chicken manure substrate. The differences among experimental groups were analyzed by one-way ANOVA followed by Duncan’s multiple range test, with * *p* < 0.05, *** *p* < 0.001.

**Figure 2:**
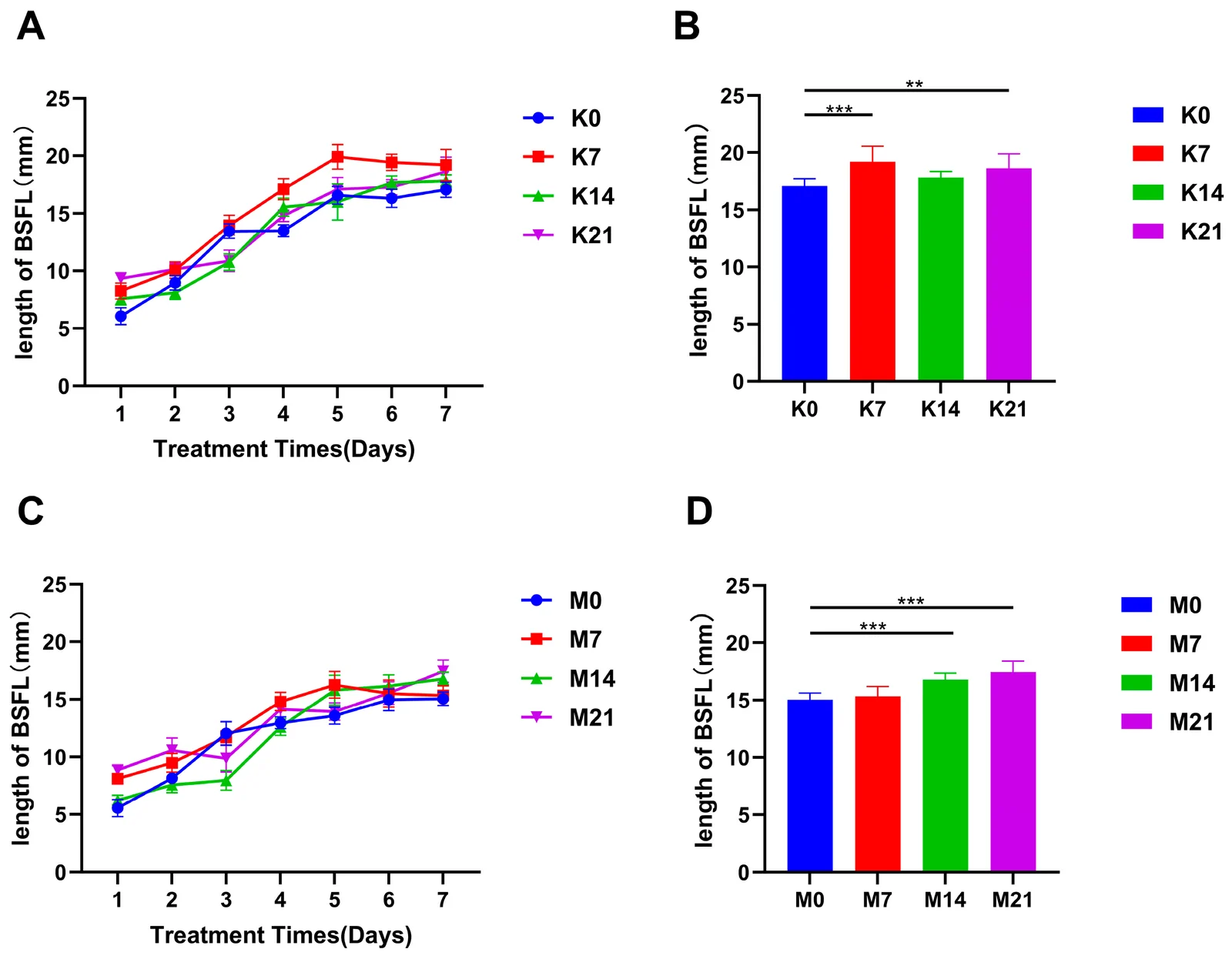
The length of BSFL raised on kitchen waste and chicken manure after different starvation times. (**A**) Daily length of BSFL on kitchen waste substrate. (**B**) Final day length of BSFL on kitchen waste substrate. (**C**) Daily length of BSFL on chicken manure substrate. (**D**) Final day length of BSFL on chicken manure substrate. The differences among experimental groups were analyzed by one-way ANOVA followed by Duncan’s multiple range test, with ** *p* < 0.01, *** *p* < 0.001.

**Figure 3:**
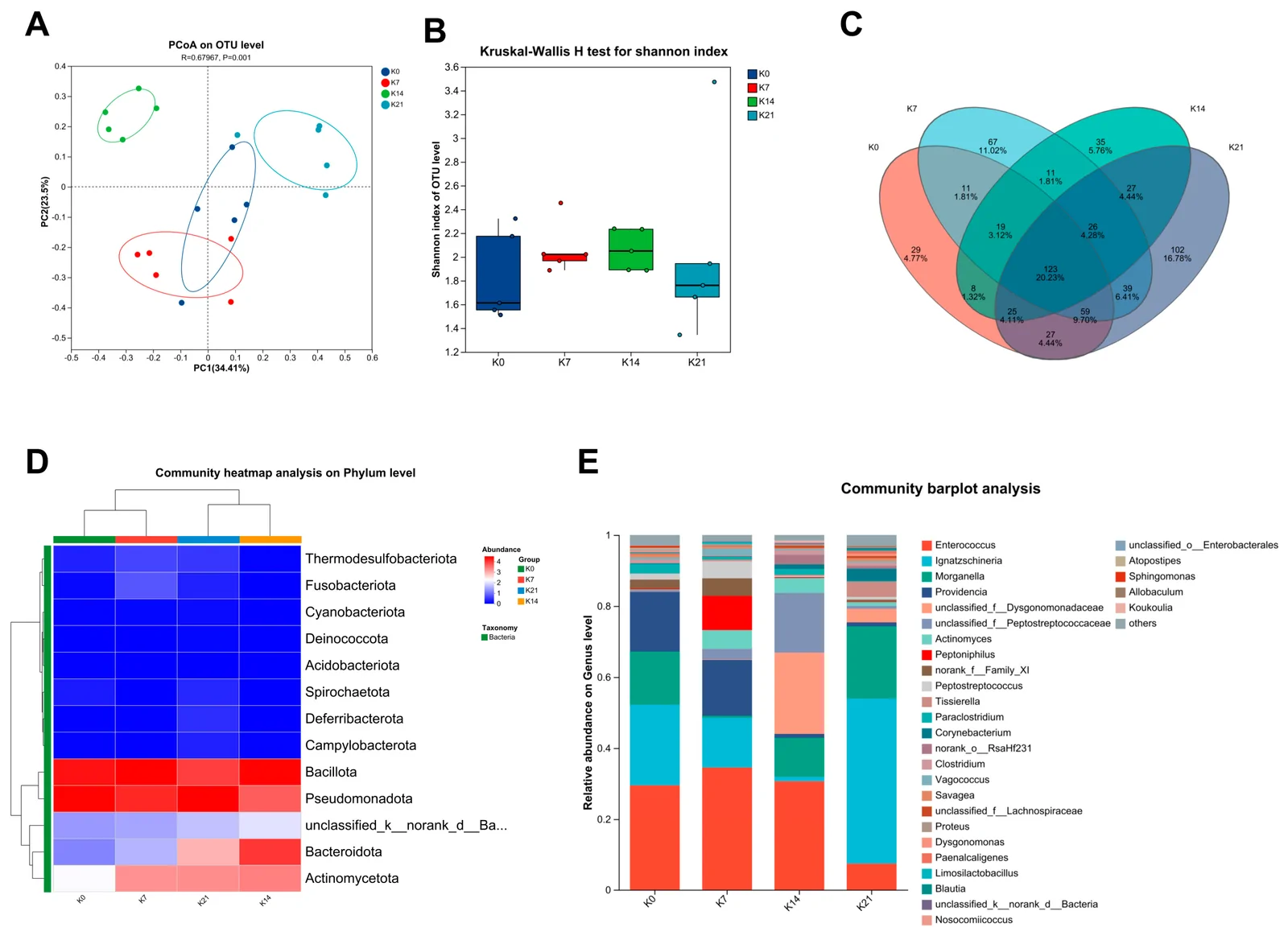
Microbiota analysis of BSFL reared on kitchen waste at different preservation times. (**A**) Principal Coordinate Analysis (PCoA) plot based on OTU-level community composition. (**B**) Box plot showing differences in Shannon index between processing groups. (**C**) Venn diagram showing the distribution of OTUs among different groups. (**D**) Heat maps of microbial community composition at the phylum level. (**E**) Bar plot of microbial community composition at the genus level.

**Figure 4:**
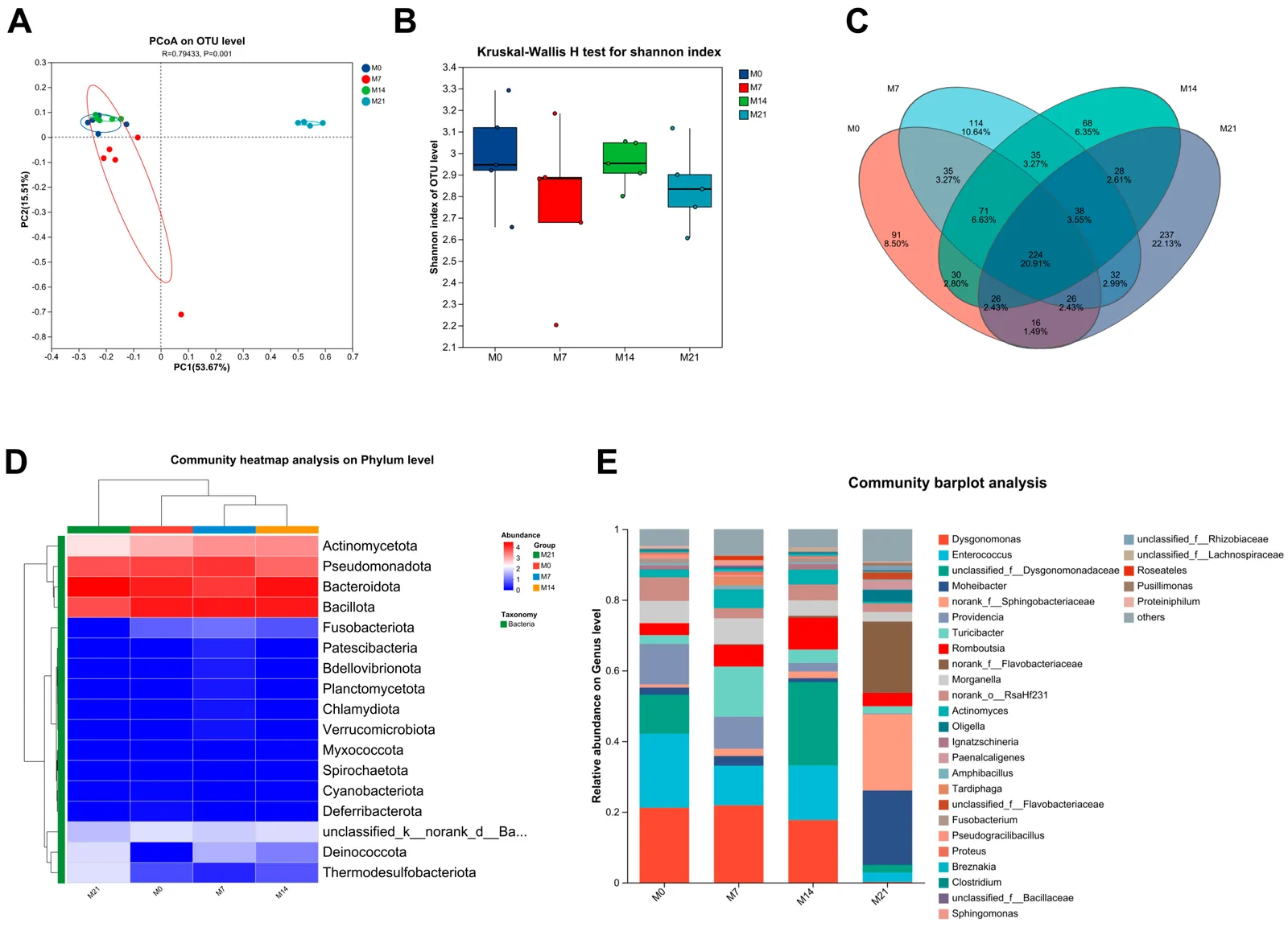
Microbiota analysis of BSFL reared on chicken manure at different preservation times. (**A**) Principal Coordinate Analysis (PCoA) plot based on OTU-level community composition. (**B**) Box plot showing differences in Shannon index between processing groups. (**C**) Venn diagram showing the distribution of OTUs among different groups. (**D**) Heat maps of microbial community composition at the phylum level. (**E**) Bar plot of microbial community composition at the genus level.

**Figure 5:**
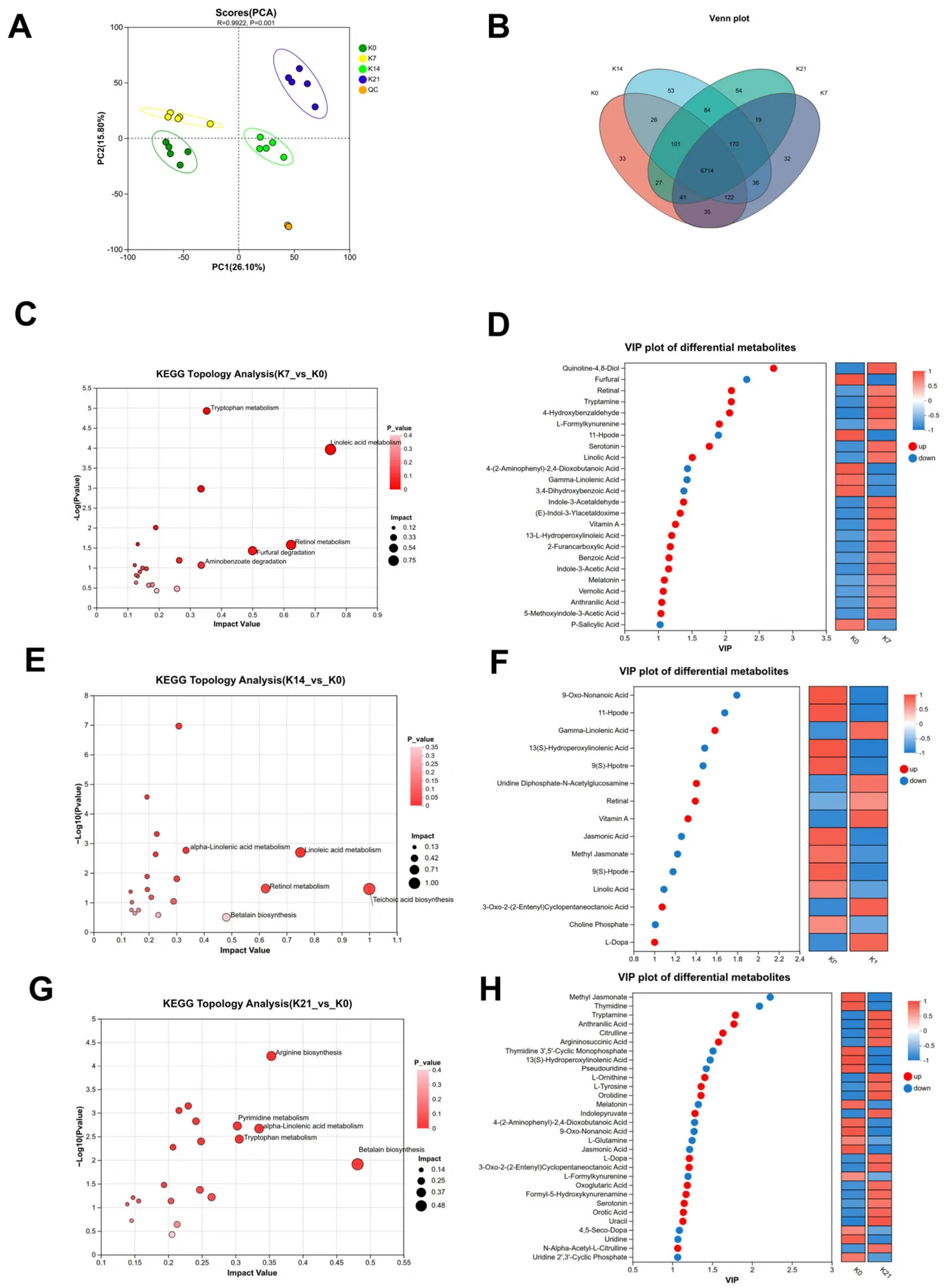
Non-targeted metabolomics analysis of BSFL reared on kitchen waste at different preservation times. (**A**) Principal Component Analysis (PCA) score plot. (**B**) Venn diagram showing the distribution of OTUs among different sample groups. (**C**,**E**,**G**) Bubble plots of KEGG pathway topology analysis for the comparison groups K7, K14, and K21 versus K0, respectively. (**D**,**F**,**H**) VIP plots of metabolites for the respective comparison groups.

**Figure 6:**
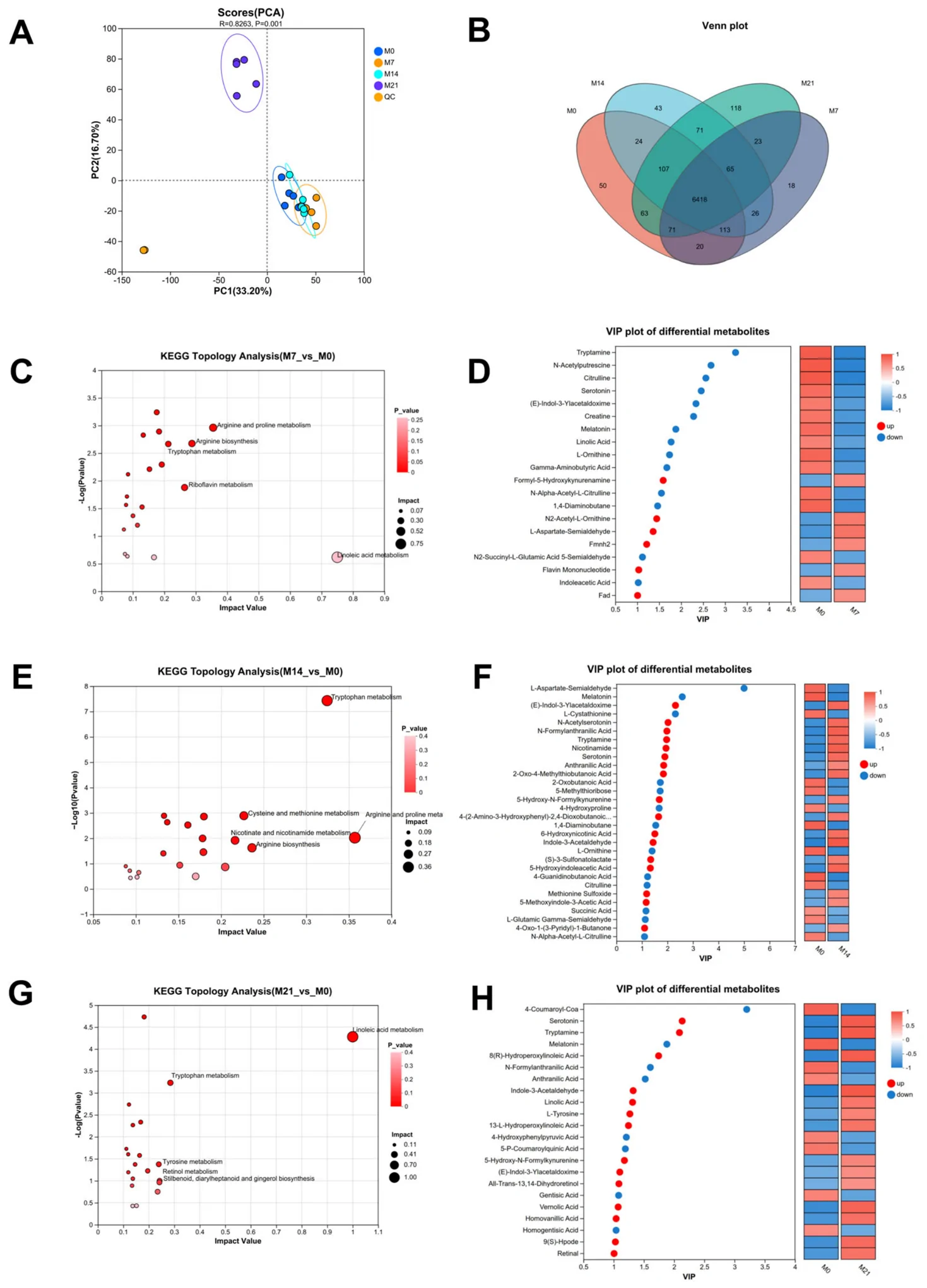
Non-targeted metabolomics analysis of BSFL reared on chicken manure at different preservation times. (**A**) Principal Component Analysis (PCA) score plot. (**B**) Venn diagram showing the distribution of OTUs among different sample groups. (**C**,**E**,**G**) Bubble plots of KEGG pathway topology analysis for the comparison groups M7, M14, and M21 versus M0, respectively. (**D**,**F**,**H**) VIP plots of metabolites for the respective comparison groups.

**Table 1:** Proximate composition of the kitchen waste and chicken manure.

Item (%)	Kitchen Waste	Chicken Manure	*p*-Value
Moisture	77.71 ± 2.01	76.05 ± 0.67	0.166
Ash	8.15 ± 2.70 ^b^	36.79 ± 3.50 ^a^	<0.001
Protein	34.62 ± 1.79	31.05 ± 4.61	0.199
Lipid	13.70 ± 0.67 ^a^	7.45 ± 0.45 ^b^	<0.001

Note: For each parameter, different letters indicate significant differences (*p* < 0.05), while the same letters or no letters indicate no significant differences (*p* > 0.05).

**Table 2:** The impact of different starvation times on the nutritional composition of BSFL reared on kitchen waste (% dry matter).

Item	K0	K7	K14	K21	SEM	*p*-Value
Moisture	60.62 ^c^	66.01 ^a^	63.20 ^b^	65.99 ^a^	0.54	<0.001
Ash	11.06 ^c^	15.02 ^a^	15.57 ^a^	12.93 ^b^	0.42	<0.001
Protein	39.60 ^c^	43.80 ^b^	46.08 ^ab^	46.60 ^a^	0.69	<0.001
Lipid	37.76 ^a^	28.11 ^c^	24.06 ^d^	33.16 ^b^	1.19	<0.001

Note: K: kitchen waste. For each parameter, different letters indicate significant differences (*p* < 0.05), while the same letters or no letters indicate no significant differences (*p* > 0.05).

**Table 3:** The impact of different starvation times on the nutritional composition of BSFL reared on chicken manure (% dry matter).

**Item**	**M0**	**M7**	**M14**	**M21**	**SEM**	***p*** **-Value**
Moisture	73.62	71.22	72.73	74.19	0.43	0.058
Ash	29.37 ^b^	30.49 ^b^	31.17 ^a^	28.31 ^b^	0.37	0.019
Protein	45.03	43.26	44.60	44.44	0.66	0.832
Lipid	4.26 ^ab^	3.64 ^b^	4.57 ^a^	4.33 ^b^	0.12	0.022

Note: M: chicken manure. For each parameter, different letters indicate significant differences (*p* < 0.05), while the same letters or no letters indicate no significant differences (*p* > 0.05).

**Table 4:** Amino acids in kitchen waste and chicken manure (% dry matter).

Amino Acid	Kitchen Waste	Chicken Manure	*p*-Value
Histidine	0.71 ± 0.02 ^a^	0.17 ± 0.01 ^b^	<0.001
Isoleucine	1.47 ± 0.03 ^a^	0.46 ± 0.02 ^b^	<0.001
Leucine	2.60 ± 0.06 ^a^	0.76 ± 0.03 ^b^	<0.001
Lysine	0.20 ± 0.01 ^a^	0.04 ± 0.00 ^b^	<0.001
Methionine	0.58 ± 0.02 ^a^	0.11 ± 0.00 ^b^	<0.001
Phenylalanine	1.42 ± 0.05 ^a^	0.43 ± 0.01 ^b^	<0.001
Threonine	1.23 ± 0.03 ^a^	0.42 ± 0.02 ^b^	<0.001
Valine	1.74 ± 0.04 ^a^	0.60 ± 0.02 ^b^	<0.001
Arginine	1.92 ± 0.04 ^a^	0.36 ± 0.02 ^b^	<0.001
EAA	11.86 ± 0.28 ^a^	3.35 ± 0.11 ^b^	<0.001
Glycine	1.56 ± 0.05 ^b^	1.88 ± 0.08 ^a^	0.004
Proline	1.39 ± 0.04 ^a^	0.55 ± 0.01 ^b^	<0.001
Aspartic	2.94 ± 0.07 ^a^	0.82 ± 0.00 ^b^	<0.001
Glutamic	5.60 ± 0.12 ^a^	1.32 ± 0.04 ^b^	<0.001
Serine	1.25 ± 0.04 ^a^	0.51 ± 0.03 ^b^	<0.001
Alanine	1.78 ± 0.04 ^a^	0.72 ± 0.02 ^b^	<0.001
Tyrosine	0.87 ± 0.01 ^a^	0.43 ± 0.03 ^b^	<0.001
NEAA	15.38 ± 0.36 ^a^	6.23 ± 0.19 ^b^	<0.001
TAA	27.25 ± 0.64 ^a^	9.58 ± 0.30 ^b^	<0.001

Note: EAA: essential amino acids. NEAA: non-essential amino acids. TAA: total amino acids. For each parameter, different letters indicate significant differences (*p* < 0.05).

**Table 5:** The effect of different starvation times on amino acid content of BSFL reared on kitchen waste (% dry matter).

Amino Acid	K0	K7	K14	K21	SEM	*p*-Value
Histidine	0.30 ^c^	0.57 ^a^	0.36 ^bc^	0.42 ^b^	0.03	<0.001
Isoleucine	1.75	1.79	1.92	2.14	0.06	0.081
Leucine	2.53 ^c^	2.81 ^bc^	2.98 ^ab^	3.24 ^a^	0.08	0.011
Lysine	2.32 ^bc^	2.11 ^c^	2.48 ^ab^	2.81 ^a^	0.07	0.002
Methionine	0.77 ^bc^	0.73 ^c^	0.86 ^ab^	0.93 ^a^	0.02	0.004
Phenylalanine	1.18 ^b^	1.85 ^a^	1.49 ^b^	1.17 ^b^	0.08	0.002
Threonine	1.22	1.22	1.39	1.32	0.03	0.109
Valine	2.26 ^b^	2.44 ^ab^	2.54 ^a^	2.69 ^a^	0.05	0.023
Arginine	1.33	1.34	1.60	1.28	0.05	0.090
EAA	13.66 ^b^	14.87 ^ab^	15.62 ^ab^	16.01 ^a^	0.33	0.039
Glycine	1.91 ^b^	2.11 ^ab^	2.22 ^a^	2.17 ^a^	0.04	0.040
Proline	2.15	2.20	2.34	2.42	0.04	0.274
Aspartic	2.53 ^b^	2.90 ^a^	3.16 ^a^	3.17 ^a^	0.08	<0.001
Glutamic	4.18	4.41	4.89	4.54	0.10	0.053
Serine	1.21	1.05	1.38	1.09	0.05	0.103
Alanine	2.66 ^b^	2.62 ^b^	2.74 ^b^	3.33 ^a^	0.08	0.003
Tyrosine	1.87	1.70	2.00	2.11	0.06	0.083
NEAA	16.49 ^b^	16.99 ^ab^	18.73 ^a^	18.94 ^a^	0.39	0.038
TAA	30.1 ^b^	31.85 ^ab^	34.35 ^ab^	34.98 ^a^	0.71	0.040

Note: K: kitchen waste. EAA: essential amino acids. NEAA: non-essential amino acids. TAA: total amino acids. For each parameter, different letters indicate significant differences (*p* < 0.05), while the same letters or no letters indicate no significant differences (*p* > 0.05).

**Table 6:** The effect of different starvation times on amino acid content of BSFL reared on chicken manure (% dry matter).

Amino Acid	M0	M7	M14	M21	SEM	*p*-Value
Histidine	0.49 ^a^	0.30 ^b^	0.36 ^b^	0.27 ^b^	0.03	0.003
Isoleucine	1.89	1.58	1.83	1.55	0.06	0.081
Leucine	2.80	2.35	2.53	2.54	0.08	0.238
Lysine	2.05	1.78	2.06	2.20	0.07	0.159
Methionine	0.53	0.44	0.55	0.58	0.02	0.152
Phenylalanine	1.72 ^a^	1.09 ^c^	1.45 ^b^	0.76 ^d^	0.09	<0.001
Threonine	1.31 ^a^	1.14 ^ab^	1.27 ^a^	1.01 ^b^	0.04	0.021
Valine	2.49	2.06	2.28	2.10	0.07	0.070
Arginine	1.60 ^a^	1.25 ^b^	1.48 ^ab^	1.02 ^c^	0.06	<0.001
EAA	14.88 ^a^	11.97 ^b^	13.80 ^ab^	12.04 ^b^	0.42	0.022
Glycine	2.36 ^a^	2.01 ^bc^	2.26 ^ab^	1.92 ^c^	0.06	0.015
Proline	2.18	1.88	2.14	1.91	0.06	0.214
Aspartic	2.88 ^a^	2.39 ^b^	2.67 ^ab^	2.41 ^b^	0.07	0.027
Glutamic	4.80 ^a^	4.03 ^bc^	4.54 ^ab^	3.82 ^c^	0.13	0.011
Serine	1.25 ^a^	1.17 ^a^	1.22 ^a^	0.81 ^b^	0.05	<0.001
Alanine	3.01 ^a^	2.40 ^b^	2.72 ^ab^	2.76 ^b^	0.08	0.026
Tyrosine	1.34	1.27	1.47	1.39	0.06	0.684
NEAA	17.82 ^a^	15.12 ^b^	17.0 ^ab^	15.02 ^b^	0.44	0.040
TAA	32.70 ^a^	27.10 ^b^	30.81 ^ab^	27.06 ^b^	0.86	0.029

Note: M: chicken manure. EAA: essential amino acids. NEAA: non-essential amino acids. TAA: total amino acids. For each parameter, different letters indicate significant differences (*p* < 0.05), while the same letters or no letters indicate no significant differences (*p* > 0.05).

**Table 7:** Fatty acid composition of the kitchen waste and chicken manure (% total fatty acids).

Fatty Acid	Kitchen Waste	Chicken Manure	*p*-Value
C10:0	0.11 ± 0.03	0.15 ± 0.01	0.154
C12:0	0.16 ± 0.02 ^b^	5.99 ± 0.49 ^a^	<0.001
C14:0	0.85 ± 0.16 ^b^	3.15 ± 0.57 ^a^	0.003
C16:0	20.35 ± 1.45	23.60 ± 2.03	0.088
C18:0	0.36 ± 0.16	0.73 ± 0.17	0.055
SFA	21.84 ± 1.46 ^b^	33.62 ± 1.98 ^a^	0.003
C16:1	1.19 ± 0.04 ^a^	1.01 ± 0.08 ^b^	0.024
*trans*-C18:1	28.26 ± 1.05 ^a^	21.43 ± 0.84 ^b^	<0.001
C20:1	4.01 ± 0.16 ^a^	1.52 ± 0.11 ^b^	<0.001
MUFA	33.47 ± 1.16 ^a^	23.96 ± 0.74 ^b^	<0.001
*trans*-C18:2	36.49 ± 0.88	37.74 ± 1.68	0.315
C18:2n-6	0.35 ± 0.04 ^b^	0.62 ± 0.03 ^a^	<0.001
C18:3	0.45 ± 0.06	0.31 ± 0.08	0.075
C20:2	0.28 ± 0.03	0.30 ± 0.03	0.459
PUFA	37.58 ± 0.78	38.98 ± 1.60	0.244

Note: SFA: saturated fatty acids. MUFA: monounsaturated fatty acids. PUFA: polyunsaturated fatty acids. For each parameter, different letters indicate significant differences (*p* < 0.05), while the same letters or no letters indicate no significant differences (*p* > 0.05).

**Table 8:** The effect of different starvation times on the fatty acid composition in BSFL reared on kitchen waste (% total fatty acids).

Fatty Acid	K0	K7	K14	K21	SEM	*p*-Value
C10:0	0.52 ^d^	0.60 ^b^	0.70 ^c^	0.92 ^a^	0.04	<0.001
C12:0	18.39 ^c^	18.40 ^c^	20.90 ^b^	26.10 ^a^	0.78	<0.001
C14:0	3.03 ^b^	3.06 ^b^	3.27 ^b^	3.86 ^a^	0.10	0.002
C16:0	12.81 ^a^	10.03 ^b^	9.48 ^b^	9.59 ^b^	0.38	<0.001
C18:0	2.67	2.48	2.19	2.31	0.07	0.096
C20:0	0.09	0.08	0.06	0.09	0.00	0.057
SFA	37.61 ^b^	34.78 ^c^	36.70 ^b^	42.97 ^a^	0.88	0.001
C14:1n-5	0.11 ^b^	0.19 ^a^	0.20 ^a^	0.18 ^ab^	0.01	0.047
C16:1n-7	2.25 ^b^	2.60 ^ab^	2.69 ^a^	2.92 ^a^	0.08	0.010
C18:1n-9c	24.68	26.39	25.34	23.94	0.39	0.145
C20:1n-9	0.07	0.08	0.07	0.11	0.01	0.059
MUFA	27.11	29.26	28.31	27.15	0.43	0.233
C18:2n-6c	29.28 ^a^	28.90 ^a^	27.58 ^a^	23.67 ^b^	0.72	0.009
C18:3n-3	2.44	2.54	2.70	2.33	0.09	0.519
C20:2n-6	0.25 ^c^	0.60 ^a^	0.50 ^ab^	0.29 ^bc^	0.05	0.016
C20:4n-6	0.47 ^d^	0.74 ^a^	0.68 ^b^	0.63 ^c^	0.02	<0.001
C20:5n-3	0.04 ^b^	0.73 ^a^	0.78 ^a^	0.06 ^b^	0.10	<0.001
C22:6n-3	0.06	0.04	0.04	0.05	0.01	0.584
PUFA	32.53 ^a^	33.54 ^a^	32.28 ^a^	27.04 ^b^	0.82	0.009

Note: K: kitchen waste. SFA: saturated fatty acids. MUFA: monounsaturated fatty acids. PUFA: polyunsaturated fatty acids. For each parameter, different letters indicate significant differences (*p* < 0.05), while the same letters or no letters indicate no significant differences (*p* > 0.05).

**Table 9:** The effect of different starvation times on the fatty acid composition in BSFL reared on chicken manure (% total fatty acids).

Fatty Acid	M0	M7	M14	M21	SEM	*p*-Value
C10:0	0.13 ^b^	0.14 ^b^	0.19 ^b^	0.32 ^a^	0.03	0.005
C12:0	3.36 ^c^	4.27 ^c^	7.03 ^b^	9.38 ^a^	0.66	<0.001
C14:0	1.37 ^c^	1.77 ^b^	2.28 ^a^	1.96 ^b^	0.09	0.001
C16:0	19.22 ^b^	22.82 ^a^	21.00 ^b^	13.02 ^c^	1.06	<0.001
C18:0	3.31 ^b^	3.98 ^a^	3.30 ^ab^	2.05 ^c^	0.24	0.006
C20:0	0.17 ^a^	0.26 ^a^	0.15 ^a^	0.11 ^b^	0.02	0.002
SFA	28.24 ^b^	33.24 ^a^	33.95 ^a^	26.83 ^b^	0.99	0.001
C14:1n-5	0.03	0.32	0.14	0.06	0.05	0.167
C16:1n-7	2.39 ^c^	2.94 ^ab^	2.51 ^bc^	3.00 ^a^	0.09	0.031
C18:1n-9c	29.05 ^a^	26.20 ^bc^	25.64 ^c^	27.33 ^b^	0.38	0.003
C20:1n-9	0.12 ^ab^	0.14 ^a^	0.07 ^c^	0.07 ^bc^	0.01	0.011
MUFA	31.59 ^a^	29.60 ^bc^	28.36 ^c^	30.46 ^ab^	0.37	0.008
C18:2n-6c	35.54 ^b^	30.26 ^d^	32.80 ^c^	38.71 ^a^	0.92	<0.001
C18:3n-3	0.65	0.59	0.62	0.66	0.03	0.789
C20:2n-6	0.45	0.42	0.36	0.31	0.02	0.052
C20:4n-6	0.31 ^ab^	0.24 ^b^	0.40 ^a^	0.26 ^b^	0.02	0.014
C20:5n-3	0.16	0.33	0.17	0.08	0.04	0.083
C22:6n-3	0.04	0.05	0.05	0.03	0.01	0.855
PUFA	37.15 ^b^	31.89 ^c^	34.40 ^c^	40.05 ^a^	0.90	<0.001

Note: M: chicken manure. SFA: saturated fatty acids. MUFA: monounsaturated fatty acids. PUFA: polyunsaturated fatty acids. For each parameter, different letters indicate significant differences (*p* < 0.05), while the same letters or no letters indicate no significant differences (*p* > 0.05).

**Table 10:** The effect of different starvation times on the production performance of BSFL reared on kitchen waste.

Item (%)	K0	K7	K14	K21	SEM	*p*-Value
SR	100 ^a^	99.20 ^a^	95.73 ^b^	67.60 ^c^	1.71	<0.001
LP	17.28 ^b^	20.20 ^a^	17.06 ^b^	15.42 ^b^	0.45	<0.001
SR_W_	86.62 ^a^	83.03 ^b^	85.74 ^ab^	72.34 ^c^	1.37	<0.001
SC_W_	19.97 ^b^	24.32 ^a^	19.89 ^b^	21.33 ^b^	0.49	<0.001
SR_D_	66.69 ^a^	69.14 ^a^	68.68 ^a^	52.52 ^b^	1.70	<0.001
SC_D_	41.88 ^bc^	50.56 ^a^	39.86 ^c^	45.08 ^b^	1.02	<0.001

Note: K: kitchen waste. SR: survival ratio. LP: larval production rate. SR_W_: substrate fresh weight reduction. SC_W_: substrate fresh weight conversion. SR_D_: substrate dry matter reduction. SC_D_: substrate dry matter conversion. For each parameter, different letters indicate significant differences (*p* < 0.05), while the same letters or no letters indicate no significant differences (*p* > 0.05).

**Table 11:** The effect of different starvation times on the production performance of BSFL reared on chicken manure.

Item (%)	M0	M7	M14	M21	SEM	*p*-Value
SR	100 ^a^	99.20 ^a^	95.73 ^b^	67.60 ^c^	1.71	<0.001
LP	9.68 ^a^	9.84 ^a^	8.20 ^b^	8.04 ^b^	0.23	<0.001
SR_W_	80.99 ^a^	78.09 ^b^	73.44 ^c^	59.60 ^d^	1.90	<0.001
SC_W_	11.96 ^b^	12.60 ^ab^	11.17 ^b^	13.49 ^a^	0.25	0.002
SR_D_	45.01 ^a^	34.51 ^b^	35.89 ^b^	27.06 ^c^	1.59	<0.001
SC_D_	24.05 ^b^	37.43 ^a^	25.18 ^b^	33.17 ^a^	1.44	<0.001

Note: M: chicken manure. SR: survival ratio. LP: larval production rate. SR_W_: substrate fresh weight reduction. SC_W_: substrate fresh weight conversion. SR_D_: substrate dry matter reduction. SC_D_: substrate dry matter conversion. For each parameter, different letters indicate significant differences (*p* < 0.05), while the same letters or no letters indicate no significant differences (*p* > 0.05).

## Data Availability

The original contributions presented in this study are included in the article. Further inquiries can be directed to the corresponding authors.
